# Happy with Your Capabilities? Valuing ICECAP-O and ICECAP-A States Based on Experienced Utility Using Subjective Well-Being Data

**DOI:** 10.1177/0272989X20923015

**Published:** 2020-05-26

**Authors:** Sebastian Himmler, Job van Exel, Werner Brouwer

**Affiliations:** Erasmus School of Health Policy Management, Erasmus University Rotterdam, Rotterdam, Zuid-Holland, The Netherlands; Erasmus School of Health Policy Management, Erasmus University Rotterdam, Rotterdam, Zuid-Holland, The Netherlands; Erasmus School of Health Policy Management, Erasmus University Rotterdam, Rotterdam, Zuid-Holland, The Netherlands

**Keywords:** capability approach, decision utility, economic evaluation, experienced utility, health state valuation

## Abstract

**Background.** The ICECAP-O and the ICECAP-A are validated capability well-being instruments. To be used in economic evaluations, multidimensional instruments require weighting of the distinguished well-being states. These weights are usually obtained through ex ante preference elicitation (i.e., decision utility) but could also be based on experienced utility. **Objective.** This article describes the development of value sets for ICECAP-O and ICECAP-A based on experienced utility and compares them with current decision utility weights. **Methods.** Data from 2 cross-sectional samples corresponding to the target groups of ICECAP-O and ICECAP-A were used in 2 separate analyses. The utility impacts of ICECAP-O and ICECAP-A levels were assessed through regression models using a composite measure of subjective well-being as a proxy for experienced utility. The observed utility impacts were rescaled to match the 0 to 1 range of the existing value set. **Results.** The calculated experienced utility values were similar to the decision utility weights for some of the ICECAP dimensions but deviated for others. The largest differences were found for weights of the ICECAP-O dimension enjoyment and the ICECAP-A dimensions attachment and autonomy. **Conclusions.** The results suggest a different weighting of ICECAP-O and ICECAP-A levels if experienced utility is used instead of decision utility.

The allocation of scarce health care resources is an important and difficult task for health care decision makers. In that context, the costs and benefits of competing health care interventions are increasingly compared with each other. Typically, such comparisons are supported by health technology assessment, with an important role for economic evaluations.^1^ In the health care decision-making context, the latter often takes the form of a cost-utility analysis in which costs are expressed in monetary terms whereas benefits are expressed in terms of quality-adjusted life-years (QALYs). Health-related quality of life is commonly measured by generic multidimensional instruments such as the EQ-5D. Health states are then valued using utility weights to create an index score anchored at 0 (dead) and 1 (full health).^1,2^

However, it has been questioned whether maximizing health, as captured in QALYs, is an appropriate representation of society’s values concerning health care^3^ or the appropriate objective in all areas of health care.^4^ The benefits of health care in many situations are not limited to health alone. In palliative and elderly care, for example, health improvement might not even represent the (primary) aim of the interventions.^5,6^ Interventions in these areas may be targeted at increasing well-being rather than health. This implies that (part of) the benefits of interventions may not be appropriately captured when using traditional health-related quality-of-life measures.

The increasing awareness of this issue has lead to the development of instruments that allow for a more complete evaluation of health care interventions. Two prominent outcome measures are the ICEpop CAPability measure for Older people (ICECAP-O) and the ICEpop CAPability measure for Adults (ICECAP-A). The ICECAP-O was developed for assessing the capability well-being of older people (65+ y).^7^ The instrument consists of 5 attributes, namely, 1) attachment, 2) security, 3) role, 4) enjoyment, and 5) control. Capability in each domain is measured using 4 levels. The ICECAP-A instrument aims to measure capability well-being in the general adult population (18+ y), using 5 dimensions: 1) stability, 2) attachment, 3) autonomy, 4) achievement, and 5) enjoyment.^4^ The validity of the ICECAP-O^8–10^ and ICECAP-A^11–13^ have been studied with generally favorable results, with the caveat that the ICECAP-O may not fully capture physical health.^14^

For use in economic evaluations, multidimensional instruments such as the ICECAP-O and ICECAP-A require not only a descriptive system of health or well-being states but also a valuation or weighting of those states. This weighting allows measured states to be expressed on a 0 (worst well-being state described with the instrument) to 1 (best well-being state described with the instrument) scale. One option to calculate such a set of weights (or tariff) is using general population preferences.^2^ The current tariffs for ICECAP-O and ICECAP-A were obtained from representative samples from the respective target populations using best-worst scaling experiments.^15,16^ These types of experiments elicit preferences by asking people to imagine being in particular states that they do not experience themselves. The obtained preference weights, therefore, are based on ex ante or decision utility.

A much debated question is whether decision utility is the appropriate basis in the context of valuing health or well-being states or whether weights should be derived from people’s experience of health and well-being states (experienced utility).^17,18^ A key advantage of using experienced utility is that weights need not be based on choices in relation to hypothetical state descriptions but can be based on the actual experience of the valued health or well-being states. Arguably, this leads to a better understanding of the effect of a health or well-being state on overall quality of life.^19^ Decision utility and experienced utility can differ substantially, with the valuation of states involving impaired physical health usually being higher when based on experienced utility,^20,21^ possibly because of coping and adaptation.^22–24^ While both decision utility and experienced utility have their advantages and disadvantages, both may be relevant for decision makers.^25^

So far, only tariffs based on decision utility are available for the ICECAP-O and the ICECAP-A well-being measures. In the large, but heterogeneous, literature regarding experienced utility-based values for health states, summarized by Cubi-Molla et al.,^26^ different approaches to assess experienced health have been proposed, including the visual analog scale or time-tradeoff using the respondent’s experienced health state. Although our current study aimed to derive tariffs based on experienced utility for broader well-being states rather than for health states, these different approaches may be relevant in that context as well, especially in deviating from deriving preferences for hypothetical states. For our current study, however, we chose a different, more direct approach to approximate experienced utility, which we deemed to be more appropriate in the context of broader well-being outcome measures. The methodology applied here entailed measuring the correlation of well-being states with subjective well-being (SWB) using regression techniques.^27,28^ This approach is derived from the notion that ratings of SWB, or life satisfaction, constitute an informative approximation of the underlying and unobservable construct of welfare or utility.^29^ To capture current experienced utility, this type of analysis requires the simultaneous measurement of SWB and the health or well-being instrument. Data provided by the two already provide relevant information on their own on the current experienced well-being state. However, combining that information to obtain an indication of the importance of the instrument’s items in terms of experienced utility arguably produces more pertinent and informative data for measuring the impact of an intervention if one is interested in what dimensions drive the outcome.

The proposed approach has been previously applied to the health state descriptive systems of the EQ-5D-3L and SF-6D using general population^30,31^ and patient data.^32^ Results indicate that differences between decision utility and experienced utility exist. The latter, for instance, gives more weight to mental health compared with pain and physical functioning, arguably because adapting to mental health problems is more difficult.^23^

This article describes the development of experienced utility tariffs for the ICECAP-O and ICECAP-A instruments based on SWB data from two general population samples from the United Kingdom. We compare our results to the existing decision utility tariffs. This information is valuable for the future use of capability well-being in health economic evaluations in contexts in which experienced and perceived capabilities are expected to diverge. We furthermore contribute to the discussion of using experienced or decision utility in economic evaluations.

## Methods

### Data

ICECAP-O and ICECAP-A were developed for the measurement of capability well-being of two different age groups (65+ y, 18+ y). Therefore, we used data from two separate cross-sectional surveys. The survey targeted at older people was administered to a sample of 516 UK citizens aged 70 y and older in 2015 and was initially designed to validate existing well-being outcome measures in the elderly.^10,14^ The adult population survey was administered to a sample of UK citizens aged 18 to 65 y in 2018. This second sample consisted of 1373 complete observations. Both surveys were intended to be representative in terms of age, gender, and education; were conducted online; and were administered by a sampling agency using quota sampling. The analysis of both instruments followed the same protocol.

### Measurement of SWB as a Proxy for Experienced Utility

We used SWB data to assess experienced utility. Our data sets contained 2 widely accepted SWB measures: Cantril’s ladder (CL) and the Satisfaction with Life Scale (SWLS). CL is a 1-dimensional instrument that asks respondents where they would place their life on a ladder ranging from worst possible to best possible life, using a 0 to 10 scale.^33^ The SWLS is a 5-item measure asking respondents to rate statements such as, “The conditions of my life are excellent” on a 7-point Likert-type scale, leading to a range of possible values from 5 to 35.^34^ While CL has the advantage of being self-anchored and intuitive, the SWLS, because of its multiple items, has higher reliability and facilitates better comparisons across individuals.^35^ No clear gold standard has been established for SWB measurement.^35^

Because of the lack of clear guidance, and because we did not want to constrain ourselves to 1 of the 2 measurements of well-being, we used a composite measure of both instruments, calculated as the unweighted averages of CL and SWLS values, which were rescaled to a 0 to 1 index.

(1) $$\mathrm{SW}B_{i}=\frac{\mathrm{SWL}S_{i}+\mathrm{Cantrils}\;\mathrm{Ladde}r_{i}}{2}$$

Such a composite measure could arguably be more robust and informative than either on its own.^36^ Although the 2 instruments are strongly related, one likely caries SWB information the other measure does not contain.^37^ In addition, combining the results of 2 instruments measuring the same concept could reduce the impact of response errors. To test the sensitivity of our results to the type of SWB measure selected, we repeated our analyses using CL and SWLS separately.

### Statistical Analysis

To estimate the relationship between ICECAP-O and ICECAP-A states and SWB, we regressed our composite measure of SWB on all levels of the 5 ICECAP dimensions for all individuals *i*. Equation (2) contains the model estimated for the ICECAP-O:

(2) $$\begin{array}{c} \mathrm{SW}B_{i}=\beta_{0}+AT_{\mathrm{il}}\beta_{\mathrm{ATl}}+\mathrm{SE}C_{\mathrm{il}}\beta_{\mathrm{SEl}}+RO_{\mathrm{il}}\beta_{\mathrm{ROl}}\\ +EN_{\mathrm{il}}\beta_{\mathrm{ENl}}+CO_{\mathrm{il}}\beta_{\mathrm{COl}}+\mathrm{SE}S_{i}\beta_{\mathrm{SES}}+\varepsilon_{i} \end{array}$$

The terms AT, SEC, RO, EN, and CO represent the vectors containing all dummy-coded levels *l* of the 5 ICECAP-O dimensions attachment (AT), security (SEC), role (RO), enjoyment (EN), and control (CO), with the highest levels of the dimensions (e.g., “I can have all the love and friendship I want”) as reference categories. SES is a vector of variables describing the socioeconomic status of individuals. This vector includes gender, age, education, marital status, financial situation, and wealth, which are expected to be related to the SWB of individuals.^38^ The model estimated for the ICECAP-A is presented in equation (3):

(3) $$\begin{array}{c} \mathrm{SW}B_{i}=\beta_{0}+ST_{\mathrm{il}}\beta_{\mathrm{STl}}+AT_{\mathrm{il}}\beta_{\mathrm{ATl}}+AU_{\mathrm{il}}\beta_{\mathrm{AUl}}\\ +AC_{\mathrm{il}}\beta_{\mathrm{ACl}}+EN_{\mathrm{il}}\beta_{\mathrm{ENl}}+\mathrm{SE}S_{i}\beta_{\mathrm{SES}}+\varepsilon_{i} \end{array}$$

ST, AT, AU, AC, and EN are vectors that contain the dummy-coded levels *l* of the ICECAP-A dimensions stability (ST), attachment (AT), autonomy (AU), achievement (AC), and enjoyment (EN), with again the highest levels of capabilities as reference categories. SES, the vector of socioeconomic variables, consists of the same variables as in the ICECAP-O model, except for replacing wealth with income, which seems more appropriate in a working-age population sample.

Equations (2) and (3) were estimated using ordinary least squares (OLS), assuming cardinality of the composite SWB values, an assumption that has been shown to hold for the type of SWB measures used in this analysis.^29^ To account for the censored nature of the SWB values (0 to 1), Tobit models were also tested. The coefficient estimates were largely similar to the OLS results, but the models were inferior concerning model fit. Functional form specifications of control variables followed model fit. A reduced model including only ICECAP-level dummies was estimated to test the robustness of ICECAP-level coefficients to model specification. Given that levels within domains have a natural order, we subjected the model to monotonicity constraints if regression results produced illogical ordering in the level coefficients. In contrast to related studies,^32^ a dummy variable indicating the worst level in any dimension was not included in the presented analysis, as the variable was not significant (*P* = 0.571 and *P* = 0.809) and did not influence coefficient estimates in either ICECAP-O or ICECAP-A regressions.

### Calculation of Tariffs

The coefficient estimates of the full models were used to construct the value sets. As the highest levels of ICECAP dimensions were taken as the reference categories in the OLS regressions, the coefficients of ICECAP-O and ICECAP-A levels represent the disutilities experienced due to being in a particular lower-capability state. The disutilities were linearly rescaled to a 0 to 1 range by summing up the level 4 coefficients, linearly extending these coefficients to sum up to 1, and multiplying the remaining coefficients with the same factor. Standard errors of the rescaled disutilities were calculated by bootstrap estimation, drawing samples with replacement and repeating the regression and rescaling steps, setting the number of bootstrap replications to 500. To test whether the disutilities were significantly different from the corresponding values based on decision utility, *t*-statistics were obtained using the calculated standard errors. The *t*-tests did not account for the uncertainty in the decision utility weights, as their standard errors were not reported.^8,15^ In a final step, the disutilities were reverse coded (e.g., the reference level was changed from “completely independent” to “unable to be at all independent”) to generate utility values, with the utility of “no capabilities” being defined as 0 (state 44444) and full capability defined as 1 (state 11111). Descriptive analysis, regressions, rescaling, and bootstrapping were performed using STATA 15.0 (Stata Statistical Software Release 15, 2018; Stata Corp, College Station, TX). Data and STATA code are available upon request from the corresponding author. The disclaimed funding source had no role in the study.

## Results

### Descriptive Analysis

Table 1 reports the characteristics of the 2 samples used for our analysis. The calculated means of CL, SWLS, and the composite SWB measure suggest that the senior population had a higher overall SWB than the sample with people aged 18 to 65 y. This result was in line with previous findings.^38^ The composite SWB measure naturally averaged out differences between CL and SWLS and had a mean of 0.66 (SD 0.19) and 0.58 (SD 0.21) for the ICECAP-O and ICECAP-A data sets, respectively.

**Table 1:** Life Satisfaction, Capabilities, and Background Characteristics of Study Samples

	ICECAP-O Data Set	ICECAP-A Data Set
Male, %	53.7	48.2
Mean age (SD), y	75.1 (4.97)	42.9 (13.7)
Finished tertiary education, %	45.2	45.4
Married, %	60.1	59.5
Make ends meet, %		
With great difficulty	4.3	8.0
With some difficulty	26.2	37.8
Fairly easy	42.3	40.0
Easily	27.3	14.2
Median household wealth, £	77,500	
Median household income per month, £		2250
Mean Cantril’s ladder score (SD)	0.70 (0.19)	0.64 (0.20)
Mean SWLS score (SD)	0.63 (0.22)	0.52 (0.24)
Mean composite SWB score (SD)	0.66 (0.19)	0.58 (0.21)
Mean ICECAP-O/-A score^a^ (SD)	0.81 (0.15)	0.75 (0.20)
*n*	516	1373

ICECAP-A, ICEpop CAPability measure for Adults; ICECAP-O, ICEpop CAPabilty measure for Older people; SD, Standard deviation; SWLS, Satisfaction with Life Scale.

a Using current decision utility value sets.

**Figure 1:**
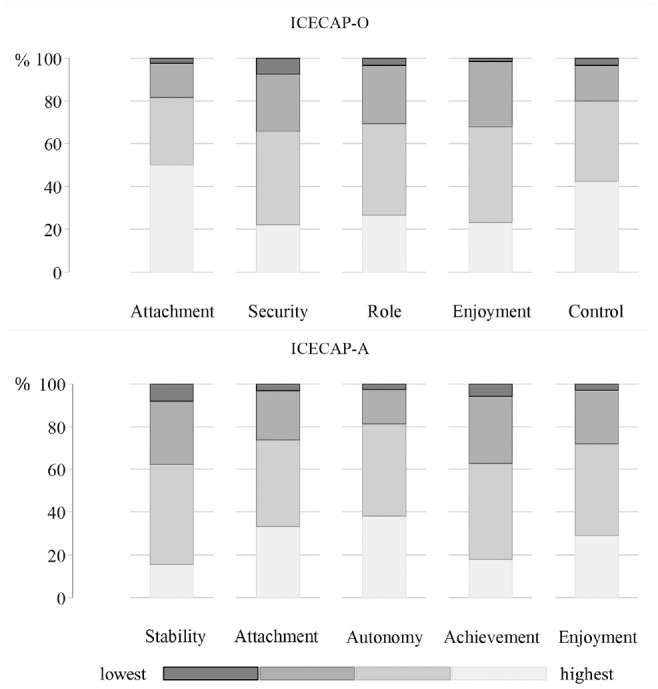
Distribution of selected capability levels per dimension in the 2 samples. ICECAP-O, ICEpop CAPabilty measure for Older people; ICECAP-A, ICEpop CAPability measure for Adults.

Figure 1 shows the distributions of selected levels per ICECAP-O and ICECAP-A dimension in both data sets. Dimensions with the lowest level of capabilities were security and enjoyment for the ICECAP-O and stability and achievement for the ICECAP-A. In all dimensions, the lowest levels of capability were selected by between 1.6% and 8.0% of respondents.

### Results from OLS Regressions

The estimation results of the impact of the ICECAP-O and ICECAP-A levels on SWB are presented in Tables 2 and 3. The tables contain both reduced models with only ICECAP levels in column I and full models including control variables in column II. Adding control variables to the 2 models only slightly changed the size of the ICECAP coefficients, whereas the improvements in *R*^2^ values from 0.628 to 0.647 and 0.630 to 0.656 were small.

**Table 2:** Impact of ICECAP-O Dimensions on Subjective Well-Being

	I: Reduced Model		II: Full Model		III: Constrained Model		IV: EU Values^a^	V: DU Values^b^	VI: Difference	*P* Value^c^
Attachment 2	−0.042**	(0.014)	−0.041**	(0.014)	−0.040**	(0.014)	−0.059	−0.021	−0.038	0.096
Attachment 3	−0.096***	(0.019)	−0.090***	(0.020)	−0.090***	(0.020)	−0.132	−0.120	−0.012	0.723
Attachment 4	−0.188***	(0.045)	−0.164***	(0.046)	−0.164***	(0.046)	−0.241	−0.266	0.025	0.683
Security 2	−0.018	(0.014)	−0.016	(0.014)	−0.016	(0.014)	−0.024	−0.072	0.048**	0.006
Security 3	−0.083***	(0.019)	−0.081***	(0.019)	−0.081***	(0.019)	−0.119	−0.113	−0.006	0.849
Security 4	−0.140***	(0.029)	−0.131***	(0.029)	−0.131***	(0.029)	−0.193	−0.147	−0.046	0.348
Role 2	0.001	(0.016)	0.004	(0.016)	0.000	(.)	0.000	−0.013	0.013	—
Role 3	−0.040	(0.026)	−0.036	(0.025)	−0.039*	(0.019)	−0.057	−0.063	0.006	0.832
Role 4	−0.096	(0.061)	−0.097	(0.062)	−0.099	(0.059)	−0.146	−0.177	0.031	0.712
Enjoyment 2	−0.062***	(0.016)	−0.058***	(0.015)	−0.057***	(0.013)	−0.083	−0.002	−0.081***	<0.001
Enjoyment 3	−0.130***	(0.023)	−0.127***	(0.023)	−0.126***	(0.021)	−0.185	−0.048	−0.137***	0.001
Enjoyment 4	−0.155**	(0.052)	−0.134*	(0.054)	−0.133*	(0.054)	−0.195	−0.149	−0.046	0.553
Control 2	−0.043**	(0.014)	−0.042**	(0.014)	−0.041**	(0.013)	−0.060	−0.025	−0.035	0.085
Control 3	−0.151***	(0.024)	−0.143***	(0.023)	−0.142***	(0.023)	−0.209	−0.102	−0.107*	0.010
Control 4	−0.146***	(0.042)	−0.154***	(0.043)	−0.153***	(0.043)	−0.225	−0.261	0.036	0.604
Male			−0.016	(0.011)	−0.016	(0.011)				
Age			0.010	(0.032)	0.010	(0.032)				
Age squared			−0.000	(0.000)	−0.000	(0.000)				
Tertiary education			−0.002	(0.011)	−0.002	(0.011)				
Married			0.029*	(0.012)	0.029*	(0.012)				
Make ends meetd										
With some difficulty			0.062*	(0.030)	0.062*	(0.030)				
Fairly easily			0.063*	(0.030)	0.064*	(0.030)				
Easily			0.097**	(0.031)	0.097**	(0.031)				
Wealth in 1,000,000 £			0.0002***	(0.000)	0.000***	(0.000)				
Constant	0.868***	(0.011)	0.327	(1.251)	0.321	(1.252)				
*n*	516		516		516					
*R* ^2^	0.628		0.647		—					

Standard errors are shown in parentheses. The highest levels of capabilities are used as reference categories. ICECAP-O, ICEpop CAPabilty measure for Older people; DU, decision utility; EU, experienced utility.

a Rescaled to 0 to 1 interval.

b From Coast et al.^15^ after reversing reference category.

c Calculated using bootstrapped standard errors.

d Reference category: with great difficulty.

* *P* < 0.05; **P < 0.01; ***P < 0.001.

The intercept coefficients for the reduced ICECAP-O and ICECAP-A models did not reach 1 (0.868 and 0.817, respectively), signaling that although capabilities describe a considerable part of SWB, full capability does not imply full SWB. On the lower end of the scale, no capabilities (ICECAP profiles 44444) corresponded to SWB of 0.143 and 0.117, respectively. Both instruments, therefore, roughly described 70% of the spread of possible SWB values.

In the full ICECAP-O model (II), shown in Table 2, all ICECAP-O levels were significant at the 5% level, except for the role dimension and the second level of the security dimension. The coefficient of the role 2 variable (i.e., role domain), answering level 2, was positive (0.004), although insignificant. To obtain consistently logical orderings, we reran the regression, constraining the role 2 variable to be zero using the STATA command cnsreg. Imposing this constraint only marginally changed the overall coefficients (III). Being married and having a better financial situation had the expected positive relationship with SWB.^38^ For ease of comparison, columns IV and V in Table 2 list the rescaled experienced disutilities of ICECAP-O levels based on SWB data as well as the decision disutilities from the above-mentioned tariffs, changing the reference category from level 5 to level 1. As column VI shows, the disutilities of the enjoyment levels were larger when calculated based on experienced utility. Further significant differences were found in a lower disutility for level 2 in the security dimension and a higher value for level 3 in the control dimension.

In the full ICECAP-A model (II), shown in Table 3, adding controls changed the coefficients of the capability levels slightly. In this model, the 3 levels of the autonomy dimension, levels 2 and 3 of the attachment dimension, and level 2 of the achievement dimension were not significant on the 5% level. The attachment levels 2 and 3 were significant in the reduced model (I), but their effect was partly absorbed by adding the controls (coefficients changed from −0.020 to −0.014 and from −0.033 to −0.024 for levels 2 and 3, respectively). Being female, married, and having less financial hardship all had an expected significant positive relationship with SWB.^38^ Comparison of disutilities based on decision and experienced utility (III and IV) using *t*-tests revealed sizable and significant differences in all ICECAP-A dimensions except for the achievement dimension (V). Higher experienced disutilities were found for the stability and the enjoyment dimensions and lower experienced disutilities for the attachment and autonomy dimensions compared with the values based on decision utility.

**Table 3:** Impact of ICECAP-A Dimensions on Subjective Well-Being

	I: Reduced Model		II: Full Model		III: EU Values^a^	IV: DU Values^b^	V: Difference	*P* Value^c^
Stability 2	−0.066***	(0.012)	−0.059***	(0.012)	−0.094	−0.031	−0.063**	0.001
Stability 3	−0.178***	(0.015)	−0.158***	(0.015)	−0.253	−0.121	−0.132***	<0.001
Stability 4	−0.251***	(0.021)	−0.219***	(0.022)	−0.351	−0.223	−0.128***	<0.001
Attachment 2	−0.020*	(0.009)	−0.014	(0.009)	−0.023	−0.039	0.016	0.234
Attachment 3	−0.033**	(0.013)	−0.024	(0.013)	−0.038	−0.131	0.093***	<0.001
Attachment 4	−0.079**	(0.028)	−0.059*	(0.026)	−0.095	−0.252	0.157***	<0.001
Autonomy 2	−0.008	(0.008)	−0.008	(0.007)	−0.013	−0.032	0.019*	0.041
Autonomy 3	−0.014	(0.012)	−0.013	(0.012)	−0.020	−0.105	0.084***	<0.001
Autonomy 4	−0.029	(0.031)	−0.025	(0.030)	−0.041	−0.182	0.141***	<0.001
Achievement 2	−0.021	(0.011)	−0.017	(0.011)	−0.027	−0.022	−0.004	0.781
Achievement 3	−0.080***	(0.014)	−0.071***	(0.014)	−0.113	−0.090	−0.023	0.310
Achievement 4	−0.171***	(0.024)	−0.159***	(0.024)	−0.255	−0.160	−0.095*	0.013
Enjoyment 2	−0.053***	(0.010)	−0.055***	(0.010)	−0.089	−0.027	−0.062***	<0.001
Enjoyment 3	−0.144***	(0.015)	−0.140***	(0.014)	−0.224	−0.112	−0.112***	<0.001
Enjoyment 4	−0.170***	(0.032)	−0.162***	(0.031)	−0.259	−0.184	−0.075	0.160
Male			−0.016*	(0.007)				
Age			0.000	(0.002)				
Age squared			0.000	(0.000)				
Tertiary education			0.003	(0.007)				
Married			0.030***	(0.008)				
Make ends meetd								
With some difficulty			0.033*	(0.016)				
Fairly easily			0.077***	(0.016)				
Easily			0.094***	(0.019)				
Monthly income in £			0.000	(0.000)				
Constant	0.817***	(0.010)	0.710***	(0.041)				
*n*	1,373		1,373					
*R* ^2^	0.630		0.656					

Standard errors are shown in parentheses. The highest levels of capabilities are used as reference categories. ICECAP-A, ICEpop CAPabilty measure for Adults; DU, decision utility; EU, experienced utility.

a Rescaled to 0 to 1 interval.

b From Flynn et al.^16^ after reversing reference category.

c Calculated using bootstrapped standard errors.

d Reference category: with great difficulty.

* *P* < 0.05; **P < 0.01; ***P < 0.001.

Supplementary Appendices A and B contain results from regressions including CL and SWLS separately, instead of a combination of the 2 SWB measures. The composite score levels out the differences between CL and SWLS coefficients, which were highest in the ICECAP-O dimensions security and role and the ICECAP-A domains attachment and autonomy. The differences in regression results were, in general, more prominent for the ICECAP-O calculations. Fewer instances of illogical orderings, fewer insignificant levels, and higher explanatory power of the models were observed when applying the composite SWB measure.

### Value Sets of ICECAP-O and ICECAP-A

The value sets based on experienced utility are presented in Table 4. The coefficients of the ICECAP levels were used for calculating the tariffs regardless of their level of significance. Analogue to the previously described findings, the largest differences compared with existing decision utility tariffs were found in the ICECAP-O dimension enjoyment and the ICECAP-A dimensions attachment and autonomy. The latter two received considerably smaller weights. Applying these experienced utility tariffs to our data changed the mean ICECAP-O utility value from 0.814 (SD 0.150) to 0.716 (SD 0.217) and the mean ICECAP-A from 0.748 (SD 0.202) to 0.656 (SD 0.238). The difference in means for the ICECAP-O can primarily be attributed to the considerably lower weights for enjoyment 2 and enjoyment 3 levels, which were selected by about 75% of respondents (Figure 1). The differences for the ICECAP-A partly had their origin in the lower values for level 1 of attachment and autonomy dimension, which represented the most frequently chosen highest capability levels in the data (Figure 1).

**Table 4:** Experienced Utility Tariffs for ICECAP-O and ICECAP-A

	ICECAP-O EU Tariff	ICECAP-O DU Tariff^a^		ICECAP-A EU Tariff	ICECAP-A DU Tariff^b^
Attachment 1	0.241	0.2535	Stability 1	0.351	0.2221
Attachment 2	0.182	0.2325	Stability 2	0.257	0.1915
Attachment 3	0.109	0.1340	Stability 3	0.098	0.1013
Attachment 4	0.000	−0.0128	Stability 4	0.000	−0.0008
Security 1	0.193	0.1788	Attachment 1	0.095	0.2276
Security 2	0.169	0.1071	Attachment 2	0.072	0.1890
Security 3	0.074	0.0661	Attachment 3	0.056	0.0964
Security 4	0.000	0.0321	Attachment 4	0.000	−0.0239
Role 1	0.146	0.1923	Autonomy 1	0.041	0.1881
Role 2	0.146	0.1793	Autonomy 2	0.028	0.1560
Role 3	0.089	0.1296	Autonomy 3	0.021	0.0836
Role 4	0.000	0.0151	Autonomy 4	0.000	0.0063
Enjoyment 1	0.195	0.1660	Achievement 1	0.255	0.1811
Enjoyment 2	0.112	0.1643	Achievement 2	0.228	0.1588
Enjoyment 3	0.011	0.1185	Achievement 3	0.142	0.0909
Enjoyment 4	0.000	0.0168	Achievement 4	0.000	0.0210
Control 1	0.225	0.2094	Enjoyment 1	0.259	0.1811
Control 2	0.165	0.1848	Enjoyment 2	0.170	0.1540
Control 3	0.016	0.1076	Enjoyment 3	0.035	0.0693
Control 4	0.000	−0.0512	Enjoyment 4	0.000	−0.0026

ICECAP-O, ICEpop CAPabilty measure for Older people; ICECAP-A, ICEpop CAPability measure for Adults; DU, decision utility EU, experienced utility.

a From Coast et al.^15^

b From Flynn et al.^16^

**Figure 2:**
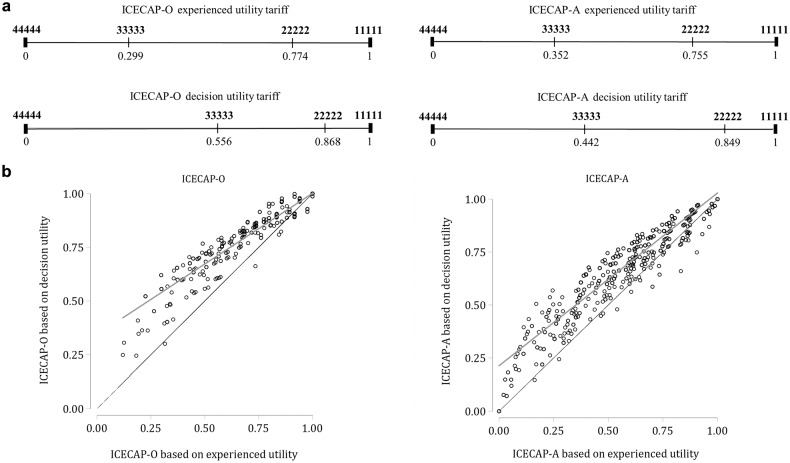
Utility values for main ICECAP profiles and sample population comparing experienced and decision utility tariffs. 44444 indicates no capabilities, 11111 full capabilities. (a) Comparison of position and index values of the main anchors (no capability, full capability) and 2 additional profiles using the 2 value sets. (b) Plot of the ICECAP index values for all observations using the 2 value sets.

Figure 2a shows the positions and ICECAP index values of 4 ICECAP profiles on the respective 0 to 1 scale applying the 2 value sets. Index scores based on experienced utility are positioned to the left of decision utility scores. The largest differences between the value sets within the 4 exemplary ICECAP profiles was found for a change from the ICECAP-O state 44444 (no capabilities) to the ICECAP-O state 33333, which increased the utility score from 0 to 0.556 using the decision utility tariffs and from 0 to 0.299 using experienced utility tariffs (i.e., a difference of 0.257). Figure 2b plots ICECAP index values for all observations used in this analysis, with experienced utility values on the *x*-axis and the decision utility values on the *y*-axis. These comparisons show that the differences between index values using the 2 sets of weights are more pronounced for lower utilities and that the discrepancy was larger for the ICECAP-O values.

## Discussion

### Summary and Context of Results

The capability instruments ICECAP-O and ICECAP-A have the potential to broaden the evaluative space of economic evaluations of health care interventions. Levels and dimensions of instruments such as the ICECAP-O and ICECAP-A have to be weighted to determine a single utility score that can be used as a measure of benefit in cost-utility analyses.^2^ These weights should ideally reflect what matters most to people and can be based on decision utility or experienced utility. This choice is not neutral, as resulting values sets can differ,^31^ as they do here. Although tariffs based on decision utility are available for ICECAP-O and ICECAP-A, this was not yet the case for experienced utility. Therefore, we developed these by directly assessing well-being capability values based on their impact on SWB using regression analysis, interpreting life satisfaction as (a proxy of) experienced utility. This is different from approaches often taken in the related literature on self-rated experienced health because of the broader nature of the ICECAP-instruments.^26^

Differences between the existing decision utility tariffs and the tariffs in general derived here were smaller for the ICECAP-O than for the ICECAP-A value sets. A surprising finding was the positive coefficient of the role 2 variable in comparison with role 1, which represented the highest level of capabilities in that domain. However, the coefficient was small (0.004) and not significantly different from zero. This finding may merely indicate little difference between level 2 and level 1 in that specific ICECAP-O dimension in terms of experienced utility. The largest differences in ICECAP-O value sets were found in the enjoyment dimension. In the ICECAP-A value set, the weights of the attachment and autonomy dimensions were considerably smaller than the decision utility weights, whereas stability and enjoyment dimensions received higher weights.

The observed differences could originate from various aspects. For instance, it could be that respondents performing decision utility exercises overestimate the impact of a specific capability domain on their utility. When occurring in real life, the impact on experienced utility may be smaller, for example, because of easier adaptation.^22,39^ Moreover, we observed relatively few people with poor capability in the attachment dimension, which may have reduced statistical power. Finally, loss of autonomy may often occur jointly with other reductions in capability, so that parts of its impact is already captured through other dimensions, which may be more pronounced in experienced utility than in decision utility, in which respondents need to consider the separate domains. One could also speculate that for some individuals, SWB may be negatively related to autonomy, as a higher level of independence might be indicative of lacking close relationships or attachment. However, we found no support for this hypothesis in our data, as we observed a positive, significant correlation between autonomy and attachment dimension (*r* = 0.25, *P* < 0.001).

To our knowledge, our study was the first to analyze the differences between valuations of capability states based on ex ante decision utility and experienced utility. The existing literature on using the latter to value health states, namely, of the EQ-5D-3L and SF-6D, shows that the estimates of the impact of specific dimensions can differ substantially between the 2 approaches,^31,32,40^ especially for mental health problems (e.g., EQ-5D dimensions anxiety and depression). The impact of mental health problems on quality of life is much smaller when based on decision utility than when based on experienced utility. One study, using experienced utility, estimated the impact of mental health problems to be about 10 times larger than the impact of mobility constraints, while these dimensions typically have similar impacts in existing tariffs based on decision utility.^31^ In that context, the discrepancies between decision and experienced utility tariffs found in our study were relatively small, in particular for the ICECAP-O.

We also emphasize that the ICECAP-O and ICECAP-A levels explain a considerably larger share of the variation in SWB (*R*^2^ of 0.63 and 0.63, respectively) than the EQ-5D or SF-6D in a previous analysis (e.g., 0.30 and 0.42 in ref. 32). The level coefficients furthermore describe a wider spread of possible SWB values than has been reported for EQ-5D and SF-6D in a similar analysis.^32^ Both are indications that the ICECAP measures indeed capture broader quality of life than just health-related quality of life.

A novelty in the approach used here is that, instead of using a single one-dimensional life satisfaction score as a proxy for SWB and experienced utility, we constructed an SWB measure based on 2 well-established measures. When replicating our analysis using the measures separately (see Supplementary Appendices A and B), we obtained similar coefficients, but the composite measure performed better than the separate measures regarding logical orderings, significant levels, and overall model fit. The use of the composite score appeared to average out differences between SWB measures and may be seen to provide a broader indication of SWB, potentially superior to using the measures separately.

An important issue worth mentioning here, although beyond the scope of the current article, is that of anchoring the value set. As mentioned, the here presented ICECAP tariffs range from the worst state described by the instrument (i.e., no capabilities, state 44444) to the best state described by the instrument (i.e., full capabilities, state 11111). Hence, the tariffs are not anchored to the state of being dead (sometimes seen as a “natural zero,” in particular for instruments measuring health). This approach is in line with the scoring of the decision utility weights of the ICECAP, which were not anchored on the state of being dead either.^7,8^ This makes it unclear how that state of being dead would relate to the scale used here and marks a clear difference with much of the QALY literature, where anchoring to the state of being dead constitutes a central concept.^2^ In general, this remains an understudied topic in well-being research and deserves attention in the future.

### Limitations

While general limitations and caveats of the chosen analytic approach are discussed elsewhere,^30,41,42^ we have to acknowledge the following limitations specific to our study. First, the data used for this study were obtained through online surveys of existing panels. Individuals participating in such online panels might differ from the general population, especially among older people. Second, our analysis is based on samples with modest sizes (516 and 1373), which, for instance, lead to relatively low numbers of observations in the lowest levels of capabilities (Figure 1). Our calculations depend on the discrepancy between coefficients of the lowest and the highest levels. Therefore, our results may be influenced by a limited number of observations regarding particular states.

Furthermore, potential endogeneity issues in our models also deserve emphasis. A reverse causal relationship between health and SWB has been shown to exist.^43^ It is not unlikely that this also the case for capability well-being, although future research using longitudinal or experimental data needs to confirm this. Our results could further be biased by omitting variables relevant to SWB. Our data set did not include variables capturing personality traits, social environment, or community involvement. All of these can be important predictors of SWB and are likely to be correlated with the level of capability well-being or their perception by individuals.^38^

Lastly, the approach we applied here is based on preferences and utility, which may, to some extent, conceptually be considered at odds with adopting the capability approach. Amartya Sen, who developed the capability approach, explicitly rejected the (exclusive) focus on emotional responses to states to determine their value, for instance, arguing that preferences adapt to circumstances and are prone to psychological biases and effects.^44^ Nevertheless, the previously established ICECAP tariffs were also based on preferences as at present there seems to be no feasible or superior alternative approaches in valuing capability well-being states.^8,15^

### Implications

The here estimated utility weights share a fair degree of similarity with the decision utility weights and more so for the better capability states than for the worse states (see Figure 2b). This creates some confidence that the chosen approach produces relevant valuations and deserves further attention. Nonetheless, aggregating the weights into specific states can produce significant differences between the 2 value sets (see Figure 2a). We do not know to what extent these differences result from the different measurement approaches or the different concepts that were measured (experienced versus decision utility). This also implies that it is unknown how the here obtained estimates relate to “true” (unobserved) underlying experienced utilities, which is also true for the existing decision utility value set. Future research could investigate and disentangle these issues further.

We reported the differences in the mean ICECAP scores applying the different tariffs (see the Results section) and, in contrast to previous analyses for health, found that the utility levels of individuals are lower when applying experienced rather than decision utility tariffs. Future studies could investigate this interesting result further, preferably in larger data sets and among patients, as one possible explanation for the current finding is that it may be driven by relatively few observations of very poor ICECAP states (see Figure 1). Furthermore, these differences and their implications should be interpreted with caution as they represent different constructs. Experienced utility incorporates coping and adaptation to well-being states,^24^ which decision utility probably does not. The presented tariffs, which were rescaled on a 0 to 1 range, tell us something about the relative weight of different levels in different dimensions, which, as such, can be compared with the relative weights from the decision utility value set. However, given that both experienced and decision utility relate to different underlying constructs, it would be inaccurate to claim that a similar absolute change based on the two tariffs indeed have the same underlying unobserved utility impact. This is because the utility scales underlying decision and experienced utility are not the same (e.g., due to adaptation).

Notwithstanding this, the comparison of value sets does highlight that a choice for either tariff set can have important consequences for evaluations. Applying the tariffs based on experienced utility would entail putting more weight on some ICECAP dimensions and less on others when assessing the benefits of an intervention as compared with using tariffs based on decision utility. Moreover, the tariffs based on experienced utility appear to result in a more even spread of the capability states on the scale. The findings shown in Figure 2a imply that the decision utility tariffs give much weight to moving people from the worst capability state (44444) to the state with poor capabilities in all domains (33333), that is, a gain of 0.556 and 0.442, respectively, for the ICECAP-O and ICECAP-A. The same improvement would be assigned a utility gain of 0.299 and 0.352, respectively, if the experienced utility tariffs were applied. These differences might have considerable implications for the assessment of interventions achieving such a change. Similarly, an improvement from state 22222 to the best capability state 11111 receives more weight when using the tariffs based on experienced utility as compared with those based on decision utility: 0.226 versus 0.132 for ICECAP-O and 0.245 versus 0.151 for ICECAP-A. Such differences highlight the importance of an informed choice on which tariffs to use to inform allocation decisions.

As this is to a large extent a normative choice, we advocate applying the here presented experienced utility-based tariffs alongside the decision utility-based tariffs for the UK context, as knowledge about the actual SWB impacts of experiencing certain states can be useful complementary information for decision making.^25^ We do advocate more research to confirm the validity of the here derived sets in that context. In general, the application of ICECAP measures as substitutes for or complements of health-related quality-of-life measures in different contexts requires further research.

Furthermore, we recommend broader use of SWB valuation approaches and presenting experienced utility as well as decision utility impacts of interventions where available and relevant. Moreover, in cases in which obtaining a value set based on decision utilities is (too) difficult or costly, the here used approach may be a reasonable and relatively straightforward alternative to produce relevant valuations of health or well-being states.

## Conclusions

Our analysis showed that calculating value sets for the ICECAP-O and ICECAP-A instruments based on experienced utility using SWB data is feasible and that the obtained weights to some extent differ from the weights previously obtained based on decision utility. This difference generates insights for policy makers in the context of the application of ICECAP-O and ICECAP-A as well as experienced and decision utility in economic evaluations.

## Supplemental Material

Appendix_A_B_online_supp – Supplemental material for Happy with Your Capabilities? Valuing ICECAP-O and ICECAP-A States Based on Experienced Utility Using Subjective Well-Being Data Click here for additional data file. Supplemental material, Appendix_A_B_online_supp for Happy with Your Capabilities? Valuing ICECAP-O and ICECAP-A States Based on Experienced Utility Using Subjective Well-Being Data by Sebastian Himmler, Job van Exel and Werner Brouwer in Medical Decision Making
