# Supplementary material for: Happy with Your Capabilities? Valuing ICECAP-O and ICECAP-A States Based on Experienced Utility Using Subjective Well-Being Data
Source: Med Decis Making. 2020 May 26;40(4):498–510. doi: 10.1177/0272989X20923015 (PMC7322999; doi:10.1177/0272989X20923015)
Supplement: Appendix_A_B_online_supp – Supplemental material for Happy with Your Capabilities? Valuing ICECAP-O and ICECAP-A States Based on Experienced Utility Using Subjective Well-Being Data [file Appendix_A_B_online_supp.docx]

**Appendix A – ICECAP-O regression results for Cantrils Ladder and SWLS separately**

|  | Compound SWB |  | Cantrils Ladder |  | SWLS |  |
| --- | --- | --- | --- | --- | --- | --- |
| Attachment 2 | -0.041^**^ | (0.014) | -0.034^*^ | (0.016) | -0.047^**^ | (0.017) |
| Attachment 3 | -0.090^***^ | (0.020) | -0.061^**^ | (0.022) | -0.119^***^ | (0.023) |
| Attachment 4 | -0.164^***^ | (0.046) | -0.141^**^ | (0.046) | -0.187^***^ | (0.056) |
| Security 2 | -0.016 | (0.014) | 0.012 | (0.016) | -0.045^**^ | (0.015) |
| Security 3 | -0.081^***^ | (0.019) | -0.039 | (0.022) | -0.123^***^ | (0.021) |
| Security 4 | -0.131^***^ | (0.029) | -0.077^*^ | (0.033) | -0.186^***^ | (0.033) |
| Role 2 | 0.004 | (0.016) | -0.001 | (0.016) | 0.008 | (0.020) |
| Role 3 | -0.036 | (0.025) | -0.036 | (0.027) | -0.035 | (0.030) |
| Role 4 | -0.097 | (0.062) | -0.147^*^ | (0.068) | -0.046 | (0.067) |
| Enjoyment 2 | -0.058^***^ | (0.015) | -0.058^***^ | (0.015) | -0.058^**^ | (0.019) |
| Enjoyment 3 | -0.127^***^ | (0.023) | -0.125^***^ | (0.025) | -0.130^***^ | (0.027) |
| Enjoyment 4 | -0.134^*^ | (0.054) | -0.165^**^ | (0.063) | -0.104 | (0.076) |
| Control 2 | -0.042^**^ | (0.014) | -0.041^**^ | (0.015) | -0.042^*^ | (0.017) |
| Control 3 | -0.143^***^ | (0.023) | -0.145^***^ | (0.028) | -0.140^***^ | (0.027) |
| Control 4 | -0.154^***^ | (0.043) | -0.140^*^ | (0.058) | -0.167^**^ | (0.051) |
| Male | -0.016 | (0.011) | -0.019 | (0.012) | -0.013 | (0.013) |
| Age | 0.010 | (0.032) | -0.005 | (0.032) | 0.026 | (0.040) |
| Age-squared | -0.000 | (0.000) | 0.000 | (0.000) | -0.000 | (0.000) |
| Tertiary education | -0.002 | (0.011) | 0.006 | (0.012) | -0.011 | (0.013) |
| Married | 0.029^*^ | (0.012) | 0.031^*^ | (0.014) | 0.026 | (0.014) |
| Make ends meet |  |  |  |  |  |  |
| with some difficulty | 0.062^*^ | (0.030) | 0.038 | (0.034) | 0.086^*^ | (0.034) |
| fairly easily | 0.063^*^ | (0.030) | 0.040 | (0.035) | 0.087^*^ | (0.034) |
| Easily | 0.097^**^ | (0.031) | 0.067 | (0.035) | 0.127^***^ | (0.035) |
| Wealth in 1,000 £ | 0.000^***^ | (0.000) | 0.000^***^ | (0.000) | 0.000^***^ | (0.000) |
| Constant | 0.327 | (1.251) | 0.968 | (1.244) | -0.313 | (1.551) |
| #illogical | 1 |  | 2 |  | 2 |  |
| #insignificant | 4 |  | 4 |  | 4 |  |
| *N* | 516 |  | 516 |  | 516 |  |
| *R*^2^ | 0.647 |  | 0.545 |  | 0.614 |  |

Note. Standard errors in parentheses; highest levels of capabilities as reference categories.

ICECAP-O, ICEpop CAPabilty measure for Older people; SWLS, Satisfaction With Life Scale.

^*^ *p* < 0.05, ^**^ *p* < 0.01, ^***^ *p* < 0.001.

**Appendix B – ICECAP-A regression results for Cantrils Ladder and SWLS separately**

|  | Compound SWB |  | Cantrils Ladder |  | SWLS |  |
| --- | --- | --- | --- | --- | --- | --- |
| Stability 2 | -0.059^***^ | (0.012) | -0.064^***^ | (0.013) | -0.054^***^ | (0.015) |
| Stability 3 | -0.158^***^ | (0.015) | -0.137^***^ | (0.016) | -0.179^***^ | (0.019) |
| Stability 4 | -0.219^***^ | (0.022) | -0.200^***^ | (0.024) | -0.238^***^ | (0.027) |
| Attachment 2 | -0.014 | (0.009) | -0.011 | (0.010) | -0.017 | (0.012) |
| Attachment 3 | -0.024 | (0.013) | -0.014 | (0.013) | -0.034^*^ | (0.016) |
| Attachment 4 | -0.059^*^ | (0.026) | -0.039 | (0.033) | -0.079^*^ | (0.031) |
| Autonomy 2 | -0.008 | (0.007) | -0.005 | (0.008) | -0.011 | (0.010) |
| Autonomy 3 | -0.013 | (0.012) | -0.014 | (0.013) | -0.011 | (0.015) |
| Autonomy 4 | -0.025 | (0.030) | -0.062 | (0.034) | 0.011 | (0.034) |
| Achievement 2 | -0.017 | (0.011) | -0.015 | (0.012) | -0.019 | (0.014) |
| Achievement 3 | -0.071^***^ | (0.014) | -0.074^***^ | (0.015) | -0.067^***^ | (0.017) |
| Achievement 4 | -0.159^***^ | (0.024) | -0.148^***^ | (0.029) | -0.170^***^ | (0.026) |
| Enjoyment 2 | -0.055^***^ | (0.010) | -0.039^***^ | (0.010) | -0.072^***^ | (0.013) |
| Enjoyment 3 | -0.140^***^ | (0.014) | -0.125^***^ | (0.016) | -0.154^***^ | (0.018) |
| Enjoyment 4 | -0.162^***^ | (0.031) | -0.186^***^ | (0.041) | -0.137^***^ | (0.037) |
| Male | -0.016^*^ | (0.007) | -0.016^*^ | (0.007) | -0.015 | (0.008) |
| Age | 0.000 | (0.002) | 0.002 | (0.002) | -0.002 | (0.002) |
| Age-squared | 0.000 | (0.000) | -0.000 | (0.000) | 0.000 | (0.000) |
| Tertiary education | 0.003 | (0.007) | 0.004 | (0.007) | 0.001 | (0.009) |
| Married | 0.030^***^ | (0.008) | 0.022^**^ | (0.008) | 0.038^***^ | (0.010) |
| Make ends meet |  |  |  |  |  |  |
| with some difficulty | 0.033^*^ | (0.016) | 0.042^*^ | (0.018) | 0.024 | (0.019) |
| fairly easily | 0.077^***^ | (0.016) | 0.076^***^ | (0.018) | 0.077^***^ | (0.020) |
| Easily | 0.094^***^ | (0.019) | 0.080^***^ | (0.021) | 0.108^***^ | (0.024) |
| Monthly income in £ | 0.000 | (0.000) | 0.000 | (0.000) | 0.000 | (0.000) |
| Constant | 0.710^***^ | (0.041) | 0.698^***^ | (0.046) | 0.723^***^ | (0.051) |
| #illogical | 0 |  | 0 |  | 3 |  |
| #insignificant | 6 |  | 7 |  | 5 |  |
| *N* | 1,373 |  | 1,373 |  | 1,373 |  |
| *R*^2^ | 0.656 |  | 0.571 |  | 0.586 |  |

Note. Standard errors in parentheses; highest levels of capabilities as reference categories.

ICECAP-A, ICEpop CAPabilty measure for Adults; SWLS, Satisfaction With Life Scale.

^*^ *p* < 0.05, ^**^ *p* < 0.01, ^***^ *p* < 0.001.
